# P38 MAPK expression and activation predicts failure of response to CHOP in patients with Diffuse Large B-Cell Lymphoma

**DOI:** 10.1186/s12885-015-1778-8

**Published:** 2015-10-16

**Authors:** Gabriel G. Vega, Alejandro Avilés-Salas, J. Ramón Chalapud, Melisa Martinez-Paniagua, Rosana Pelayo, Héctor Mayani, Rogelio Hernandez-Pando, Otoniel Martinez-Maza, Sara Huerta-Yepez, Benjamin Bonavida, Mario I. Vega

**Affiliations:** 1 Oncology Research Unit, Oncology Hospital, Siglo XXI National Medical Center, IMSS, Mexico City, Mexico; 2 Facultad de Medicina Programa de Posgrado, Doctorado en Ciencias Biomédicas UNAM, México City, DF Mexico; 3 Departamento de Patología, Instituto Nacional de Cancerología, SSA, México City, México; 4 Servicio de Hematología, Instituto Nacional de Cancerología, SSA, México City, México; 5 Unidad de Investigación Médica en Inmunología e Infectología, CMN La Raza, IMSS, México City, México; 6 Departamento de Patología Experimental, Instituto Nacional de Ciencias Médicas y Nutrición, Salvador Zubiran, SSA, México City, México; 7 Department of Microbiology, Immunology and Molecular Genetics, David Geffen School of Medicine UCLA, Los Angeles, CA USA; 8 Unidad de Investigación en Enfermedades Oncológicas, Hospital Infantil de México, Federico Gómez, SSA, México City, México

**Keywords:** DLBCL, p38 MAPK, Prognosis, Survival, CHOP, I120

## Abstract

**Background:**

The p38 MAPK is constitutively activated in B-NHL cell lines and regulates chemoresistance. Accordingly, we hypothesized that activated p38 MAPK may be associated with the *in vivo* unresponsiveness to chemotherapy in B-NHL patients.

**Methods:**

Tissue microarrays generated from eighty untreated patients with Diffused Large B Cell Lymphoma (DLBCL) were examined by immunohistochemistry for the expression of p38 and phospho p38 (p-p38) MAPK. In addition, both Bcl-2 and NF-κB expressions were determined. Kaplan Meier analysis was assessed.

**Results:**

Tumor tissues expressed p38 MAPK (82 %) and p-p38 MAPK (30 %). Both p38 and p-p38 MAPK expressions correlated with the high score performance status. A significant correlation was found between the expression p-p38 and poor response to CHOP. The five year median follow-up FFS was 81 % for p38^−^ and 34 % for p38^+^ and for OS was 83 % for p38^−^ and 47 % for p38^+^. The p-p38^+^ tissues expressed Bcl-2 and 90 % of p-p38^−^ where Bcl-2^−^. The coexpression of p-p38 and Bcl-2 correlated with pool EFS and OS. There was no correlation between the expression of p-p38 and the expression of NF-κB.

**Conclusion:**

The findings revealed, for the first time, that a subset of patients with DLBCL and whose tumors expressed high p-p38 MAPK responded poorly to CHOP therapy and had poor EFS and OS. The expression of p38, p-p38, Bcl2 and the ABC subtype are significant risk factors both p38 and p-p38 expressions remain independent prognostic factors.

**Electronic supplementary material:**

The online version of this article (doi:10.1186/s12885-015-1778-8) contains supplementary material, which is available to authorized users.

## Background

The term Diffuse Large B-Cell Lymphoma (DLBCL) is applied to a heterogeneous group of clinically aggressive disorders that collectively account for more than 30 % of all new lymphoma diagnoses. DLBCL is the most common type of Non-Hodgkin Lymphoma (NHL) that occurs in the western world, with different clinical, immunophenotypic, and genetic abnormalities [1]. DLBCL is associated with an aggressive natural history and a median survival of less than one year in untreated patients [2]. The incidence of NHL increased dramatically from the 1970’s until 1990’s with an estimated of over 60,000 new cases diagnosed in the United States and around 30,000 in Mexico in 2011 [2]. Patients who are diagnosed with DLBCL must undergo full stating work-up which will determine the treatment schedule and that may help to identify prognostic factors [3]. The International Prognostic Index (IPI), proposed in 1993, is still the primary clinical tool used to predict DLBCL outcomes [4]. However, the IPI was developed prior to the rituximab (anti-CD20 mAb) era and has recently been the subject of modifications for a better prediction of the outcome [5].

Two biological distinct pathophysiologic entities of DLBCL -as determined by gene expression profile-, namely, the Germinal Center B-cell subtype (GCB) and the Activated B-cell (ABC) subtype, and unclassified subtypes, derived from different cells of origin [6]. Patients with GCB-DLBCL have a better survival span than those with ABC-DLBCL [7]. Although the use of rituximab has improved the overall survival rates [8], however, a survival advantage in patients with GCB-DLBCL persists.

A number of prognostic markers have been suggested for DLBCL, including p53, Myc, Bcl-2 and NF-κB [9]. However, different studies have shown divergent results and the prognostic values for some of the markers are still unclear. P38 MAPK (Mitogen-Activated Protein Kinase) belongs to a class of serine/threonine kinases [10] composed of four isoforms (α, β, γ and δ). P38 α and p38 β MAPK are expressed in most tissues [11]. Phosphorylation of p38 activates a wide range of substrates that include transcription factors, protein kinases, cytosolic and nuclear proteins, all of which lead to diverse responses such as inflammation, cell differentiation, cell-cycle arrest, apoptosis, senescence, cytokine production and regulation of RNA splicing.

The role of p38 in cancer may depend on the cell type and cancer stage. Some studies have reported that p38 MAPK increases tumor cell proliferation whereas in others the activation of the p38 MAPK pathway results in tumor suppression [12]. It remains to be determined how these two opposing functions of p38 MAPK –mediated signaling operate *in vivo* and how they may impact cancer development [13]. Increased levels of activated p38 MAPK (phospho (p)-p38 MAPK) have been correlated with malignancy in follicular lymphoma and other malignancies [14]. Our reported studies in B-NHL cell lines have shown that treatment with rituximab inhibited p-p38 MAPK expression, reduced Bcl-2 expression, and chemosensitized such resistant cell lines to drug-induced apoptosis [15]. Hence, the activation of the p38 MAPK pathway has emerged as a major mechanism of rapid cell proliferation and tumor cell resistance to cytotoxic drugs [16]. The expression and activity of p38 MAPK have been demonstrated to be key players in the cellular response to several drugs used in cancer therapy [17].

Since the standard treatment to DLBCL includes rituximab (R), several studies have been reported with conflicting observations concerning the prognosis of CHOP versus R-CHOP-treated patients [18]. Interestingly, in some countries of Latin-America, including Mexico, some patients have no access to treatment with R-CHOP and the usual treatment regimen for them is just CHOP. Hence, we were interested to determine which subset of patients with DLBCL may respond to treatment with CHOP alone.

Based on our findings that the activation of p38 MAPK renders the tumor cells chemo-resistant [15], we hypothesized that the expression and activation of p38 MAPK (p-p38 MAPK) may be inversely correlated with the response to CHOP treatment in patients with DLBCL. The present study was designed to test this hypothesis and the followings were investigated: 1) the expression levels of p38 MAPK and p-p38 MAPK in a cohort of tumor tissues derived from untreated DLBCL patients who were subsequently treated with CHOP 2) the determination whether the p38 MAPK and/or p-p38 MAPK expression level had a prognostic significance in the context of treatment in response to CHOP and 3) the assessment of the predictive value of p-p38 MAPK expression as a biological marker for the unsuccessful treatment with CHOP in patients with DLBCL that cannot be treated with R-CHOP. The findings corroborated the above hypothesis.

## Methods

### Patients and Tumor Specimens

We have retrospectively studied 80 tumor tissues from DLBCL patients derived from the National Cancer Institute (Instituto Nacional de Cancerología INCan, SSA, Mexico City), who subsequently received the CHOP (Cytoxan, Doxorubicin, Vincristine and Prednisone)-based chemotherapy regimen as first-line chemotherapy. The patients studied fulfilled the following criteria: a) Histopathological diagnosis of DLBCL according to the WHO classification, b) the samples excluded patients who were treated with CHOP plus rituximab and c) the samples were from patients with no human immunodeficiency virus-infection. Cases with any confirmed follicular architecture were not eligible for the study. The disease stage was evaluated according to the Ann Arbor staging system [19]. The scoring system for Performance Status evaluation was according to the Eastern Corporative Oncology Group (ECOG) [20]. The clinical parameters of the studied patients are summarized in Table 1. The tumor response was evaluated according to the WHO recommendations after 6 cycles of chemotherapy. Fifty-two (65 %) patients showed completed remission after CHOP therapy. This retrospective study was approved by the National Commission for Scientific Research, the Ethics Board of the Mexican Institute for Social Security (IMSS 2006-785-048) and it is in accordance with the Declaration of Helsinki. A written informed consent was obtained from each patient and all lymphoma biopsies had been offered according to National Commission for Scientific Research of the IMSS (CIS) guidelines.

**Table 1:** Clinical parameters of patients with DLBCL

Clinical parameters	Frequency n (%)
Gender	
Male	45 (56)
Female	35 (44)
Age	
Media, range	55.15–85
Stage	
I-II	28 (35)
III -IV	52 (65)
LDH	
Normal	42 (52)
High	38 (48)
Performance Status	
0–1	60 (75)
2–4	20 (25)
IPI	
0–2	57 (72)
3–5	23 (28)
Bcl-2	
Positive	30 (37)
Negative	50 (63)
NF-kB	
Positive	26 (32)
Negative	54 (68)
Phenotype	
GCB	58 (72)
ABC	22 (28)

The IPI was defined on the basis of five clinical variables (ages, performance status, stage, LDH level and extranodal site involvement) and subdivided into IPI groups as previously reported [21]: a low IPI score (0–2) and a high IPI score (3–5).

Tumor tissues were fixed and embedded in paraffin for routine histological and immunohistochemical studies at diagnosis. They were re-evaluated by two pathologists (A A-S & R H-P) and the histological diagnosis was established according to the current REAL/WHO criteria. The pertinent clinical information of these patients was obtained by reviewing the tumor registry records and/or from the patient’s medical charts.

### Immunohistochemistry

A tumor microarray (TMA) from the 80 DLBCL patients was constructed as previously described [22], using 3 malignant tissue cores (0.5 mm diameter) per tumor. The TMA sections were incubated overnight at room temperature with ether anti-p38 or anti-p-p38 MAPK (Thr180/Tyr182; Santa Cruz) antibodies. Standard Immunohistochemical analysis was carried out for Bcl-2 as previously reported [23]. The optimal cut-point for Bcl-2 expression was determined by the method used by Iqbal et al. [23]. For NF-κB analysis, a rabbit polyclonal anti-NF-κB p65 of human origin (NF-κB (C-20), Santa Cruz Biotechnology) was used as previously reported [24]. A standard detection system was then used (Universal LSAB kit DAKO Corporation, Carpinteria, CA, USA). The slides were then analyzed under light microscopy (Olympus BX-40).

The GCB and ABC DLBCL subtypes were defined based on the CD10, Bcl-6 and MUM-1 staining and according to the algorithm developed by Hans et al. [22]. The GCB type was defined as CD10^+^ or CD10^−^/Bcl-6^+^/MUM-1^−^, whereas the ABC subtype was defined as CD10^−^/Bcl-6^−^or CD10^−^/Bcl-6^+^/MUM-1^+^. The Additional file 1: Table S1 summarizes the various antibodies used for analysis.

Semi-quantitative assessment of tissue antibody staining was conducted by an expert pathologist on lymphoma analysis (A A-S), who was blinded to the pathological variables. The stained slides were verified by a second expert to ensure consistency in scoring (R H-P). The expression levels on the TMA’s were done blindly. The TMA spot was a second blinded quantitative assessment by the same pathologist. The target tissue for scoring was done in the malignant cells by considering the nuclear and cytoplasmic staining pattern for p38 and p-p38. Data are presented as positively stained target cells per 100 cells (range 0–100 % positive), per tissue region in the TMA (4 regions in each TMA spot). We have calculated the percentage of positive staining by using the thresholds of ≤ 30 % of stained cells as negative. Stained cells that are ≥ 30 % were considered as positive. All of the results are expressed according to these calculations. To establish the positivity and the threshold (30 %), we categorized by analysis as defined by the ROC curves and by the Younden index in order to separate the percentages depending on the response to treatment. Overall differences were compared by the log-rank test. The tumor response was evaluated according to the WHO recommendations after each of the 6 cycles of chemotherapy.

### Statistical analyses

Survival curves were estimated by the Kaplan-Meier method with differences compared by the log-rank test [25], whereby the event-free survival (EFS) and the overall survival (OS) were considered the main outcomes of the study. The EFS was calculated from the date of diagnosis to the date of documented disease progression, relapse or death from any cause. The OS was calculated from the date of diagnosis until death from any cause or the last follow-up. Comparisons of the clinical characteristics between the p38 MAPK and p-p38 MAPK-positive and negative groups were determined by the Fisher’s exact test and by the Student’s *t*-test. Cox multivariate analysis was carried out to estimate the prognostic impact of the biomarkers and IPI risk factors in both the EFS and the OS. The data were analyzed using the Graph Pad Prism-5 software for windows (La Jolla, CA).

## Results

### Patient characteristics

In this study, 80 patients with DCBCL that were treated with CHOP at the National Institute of Cancer (INCan, SSA, Mexico) between 2000 and 2005 were analyzed. The clinical characteristics of those patients are listed in Table 1. Twenty three (28 %) patients were included in the high IPI group. Twenty (25 %) patients were included in the high (2–4) Performance Status. Bcl-2 was expressed in 30 (37 %) patients. None of the Bcl-2^+^ patients had history of other lymphoproliferative disorders. Fifty eight patients (72 %) were categorized into the GC subtype and 22 (28 %) into the ABC subtype. Twenty six patients (32 %) expressed p65 and p50 NF-κB and 13 patients (16 %) expressed p50 NF-κB. (Additional file 2: Figure S1). Fifty two patients showed a disappearance of all evidence of disease and negative PET and were classified as responders (R). The remaining 28 patients, with any new lesion or an increase by ≥ 50 % of previously involved sites, were classified as non-responders (NR).

### Clinical and biological features in relation to p38 MAPK and p-p38 MAPK expressions

**Fig. 1:**
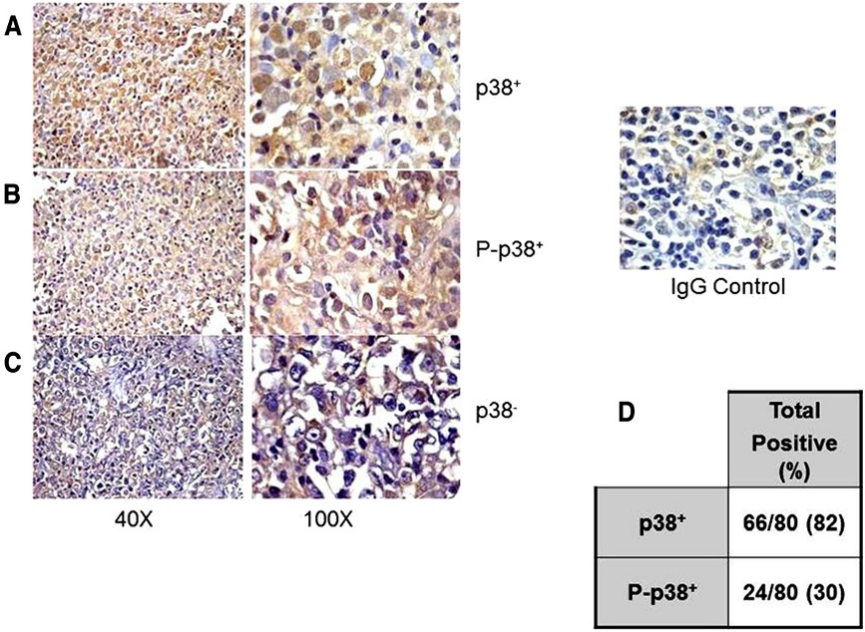
Expression of p38 MAPK and p-p38 MAPK in patients with DLBCL Tissue microarray-based immunohistochemical analyses of p38 MAPK and p-p38 MAPK in representative tumor biopsies from DLBCL patients. DLBCL array cores showing over-expression of p38 MAPK (**a**), high expression of p-p38 MAPK (**b**) and another DLBCL tissue array core shows no or low expression of p38 MAPK (**c**). 40X objective on an Olympus BX 51 microscope (Olympus America, Center Valley, PA, USA), (Left) and 100X aperture view of the same tissue (Right). Of the 80 DLBCL biopsies analyzed, 82 % were positive for p38 and only 30 % were positive for p-p38 MAPK (**d**). The percentages were calculated based on the threshold used for ≤ 30 % expressed cells considered negative and ≥ 30 % stained cells were considered positive as descried in methods

The expressions or lack of expressions of p38 MAPK and p-p38 MAPK in representative samples are shown in Fig. 1. Figure 1a shows the detection of p38 expression in tumor cells. Figure 1b shows the detection of p-p38 expression in tumor cells and Fig. 1c shows the absence of detection of p38. The IgG isotype was used as a specificity control and is shown on the right panel. The analysis of the expression pattern in all of the tumor specimens showed that 66/80 (82 %) of tumor tissues were p38^+^ and 24/80 (30 %) were p-p38^+^ (Fig. 1d). The expression patters of p38^+^, p38^−^, p-p38^+^, and p-p38 ^−^ were analyzed as a function of the patients clinical characteristics. No significant differences were observed among the above four subgroups with regards to the distribution of patients characteristics including the IPI score (67 % vs 50 % High IPI 3-5/p-p38, *p* > 0.5; 56 % vs 57 % High IPI 3-5/p38, *p* > 0.5), stage of disease (67 % vs 64 % with stage III-IV disease/p-p38, *p* > 0.4; 67 % vs 57 % with stage III-IV disease/p38, *p* > 0.4), lactate dehydrogenase (LDH; 50 % vs 67 % with elevated LDH/p-p38, *p* > 0.3; 61 % vs 71 % with elevated LDH/p38, *p* > 0.3). However, there were more p-p38 expression in patients with poor PS (*p* < 0.01) (Table 2). These results suggested that high p-p38 expression correlated with a poor performance status and can have a greater impact on an individual’s prognosis. The ABC subtype was also influenced by higher p-p38^+^ (92 %) and p38^+^ (34 %) (*p* < 0.01). In the GC subtype, only 2/24 (8 %) were p-p38^+^ and 44/66 (66 %) were p38^+^ (*p* < 0.05).

**Table 2:** Patients characteristics in relation to p38 MAPK and p-p38 MAPK expressions

Features	p-p38^+^ (n = 24), n (%)	p-p38^-^ (n = 56), n (%)	p38^+^ (n = 66), n (%)	p38^-^ (n = 14), n (%)	*p*
Sex:					
Male	16 (64)	29 (52)	40 (61)	6 (42)	1.0
Female	8 (36)	27 (48)	26 (39)	8 (58)	
Age: Media, range	55, 30–65	60, 25–72	46, 20–68	53,15–75	0.74
Stage:					
1–2	8 (33)	20 (36)	22 (33)	6 (43)	0.26
3–4	16 (67)	36 (64)	44 (67)	8 (57)	0.44
LDH:					
Normal	15 (50)	27 (33)	30 (39)	12 (29)	0.38
High	9 (50)	29 (67)	36 (61)	2 (71)	0.33
IPI					
0–2	19 (33)	38 (50)	47 (44)	10 (43)	0.31
3–5	5 (67)	18 (50)	19 (56)	4 (57)	0.51
PS > 1	*8 (33)	*5 (9)	*17 (26)	*1 (7)	* < 0.01
Bcl-2					
Positive	*24 (100)	*6 (10)	29 (45)	7 (50)	*0.003
Negative	*0 (0)	*50 (90)	37 (55)	7(50)	*0.007
GC type	‘2 (8)	*56 (100)	*44 (66)	*14 (100)	* < 0.05
ABC	*22 (92)	*0 (0)	*22 (34)	*0 (0)	* < 0.01
NF-kB					
Positive	13 (54)	26 (46)	26 (40)	6 (42)	0.82
Negative	11 (46)	30 (54)	40 (60)	8 (57)	0.96

*IPI* International Prognostic Index, *LDH* Lactate Dehydrogenase, *PS* Performance status, *GC* germinal Center, *ABC* Activated B-Cell

For NF-κB expression, there were no significant differences observed among the four subgroups according to p-p38 or p-38 expression. Seventy-seven percent of patients with p-p38^−^ were also negative for NF-κB and 70 % of patients with p38^+^ were negative for NF-κB. There was no correlation between these groups (Table 2). The predictive role of p-p38 expression was evaluated by using ROC analysis, which was performed by estimating the percentage of p-p38 expression according to treatment response and all the patients with over 30 % were established as positive for p-p38 expression (data no shown).

The above findings demonstrated that both the PS and the ABC subtype of tumor tissues exhibited high p-p38 expression.

### Relationship between the p38 and p-p38 expressions in tumor tissues and the response of patients CHOP therapy

**Fig. 2:**
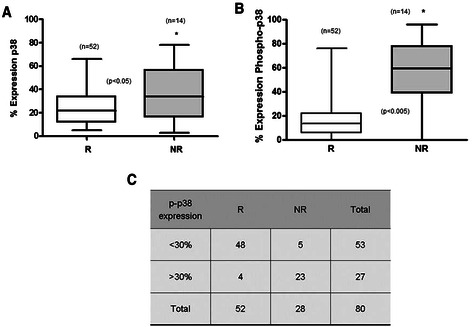
p38 MAPK and p-p38 MAPK expressions in non-responders DLBCL patients. **a** p38 MAPK expression according to the clinical response. P38 MAPK expression was assessed by IHC in 80 biopsies. The % expression was calculated from the TMA p38 MAPK staining. Black lines represent the medians, whiskers marking the lower and upper adjacent values. **b** p-p38 MAPK expression in responder (R) and non-responder (NR) patients. The % of tumor samples expressing p-p38 was determined from the TMA staining of p-p38 MAPK. **c** There is a significant expression of p-p38 MAPK in the NR. The % of p-p38 MAPK expression was determined in TMA and the % threshold (30) was defined by the ROC curves and by the Younden index to separate the % depending on the response to treatment

Fifty two (65 %) of the 80 patients examined in this study were classified as responders (R) and 28 (35 %) were classified as non-responders (NR) to CHOP treatment. A comparison between the R and NR for the expression of p38 demonstrated that the NR patients showed higher expression of p38 than found in the R patients (Fig. 2a) (*p* < 0.05). Noteworthy, the expression of p-p38 was significantly higher in the NR patients than in the R subgroup of patients (Fig. 2b) (*p* < 0.005). Figure 2c summarizes the findings for p-p38 expression and demonstrate that the majority of tumor tissues from the NR showed higher expression of p-p38, suggesting that p-p38 expression may be involved in the resistance to CHOP treatment. The correlation between high p-p38 expression and poor response to CHOP treatment is significant (Fig. 2c).

### Clinical outcomes according to the expression of p38 and p-p38 MAPK

**Fig. 3:**
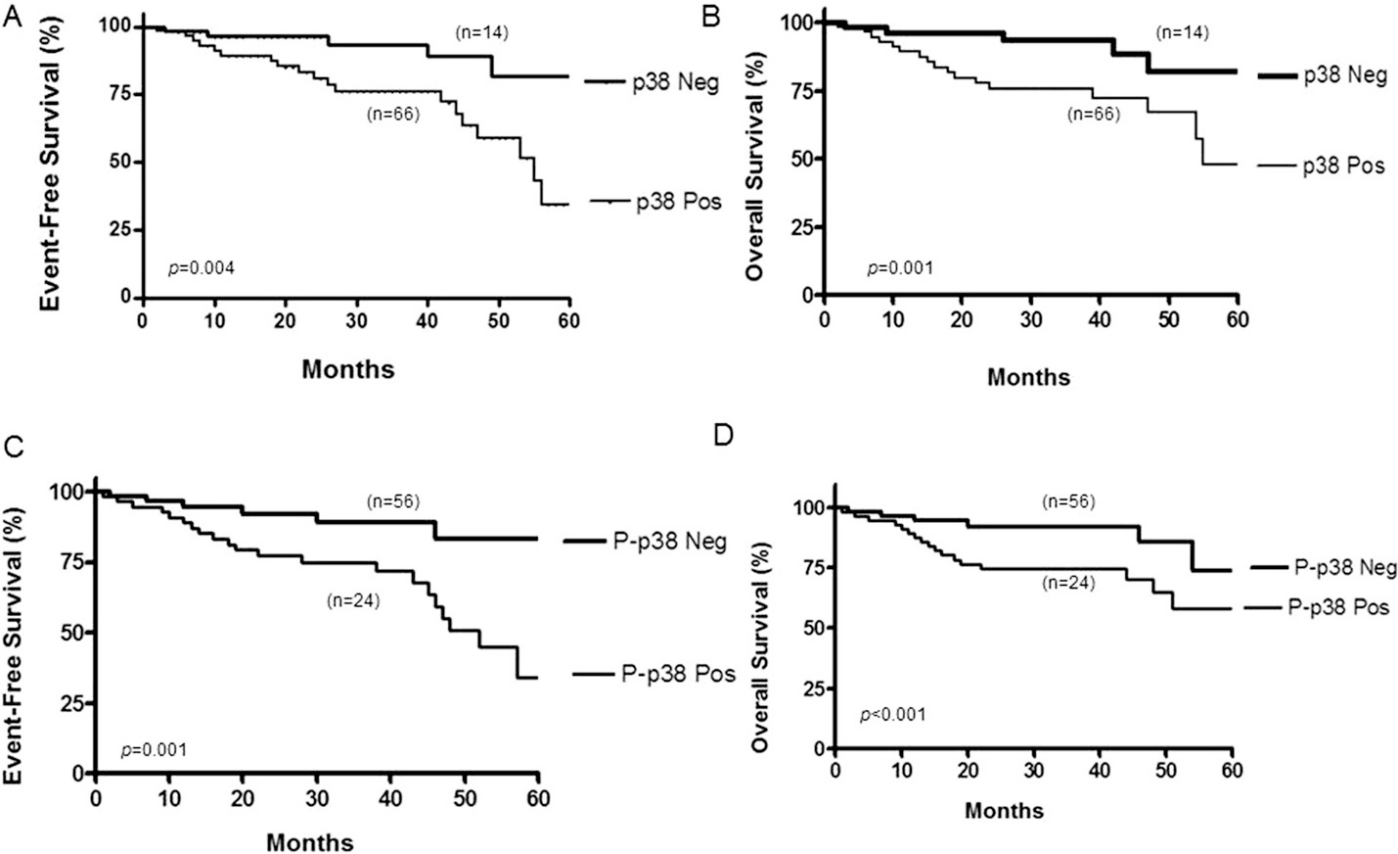
Impact of p38 and p-p38 expressions on the prognosis of DLBCL patients. **a** Event-free survival (EFS) curves for DLBCL patients treated with CHOP according to p38 MAPK expression. The curves correspond to positive (*n* = 66) versus negative (*n* = 14) p38 MAPK expression **b**. Overall survival (OS) curves for DLBCL patients treated with CHOP according to p38 MAPK expression. **c** EFS curves according to the p-p38^+^ MAPK expression (*n* = 24) versus p-p38-MAPK (*n* = 56) **d**. Overall survival (OS) curve for DLBCL patients treated with CHOP according to positive p-p38 MAPK expression (*n* = 24) versus p-p38^−^ MAPK expression (*n* = 56)

We examined the clinical outcomes of patients based on p38 expression by the Kaplan-Meier analysis. After a median follow-up of 60 months, the EFS was 81 % for the p38^−^ tumor tissues and 34 % for the p38^+^ tumor tissues (*p* = 0.004) (Fig. 3a). Further, the 5-year OS was 83 % for the p38^−^ tumor tissues vs 47 % for the p38^+^ tumor tissues (*p* = 0.001) (Fig. 3b). These findings demonstrated the impact of p38^+^ expression in tumor tissues on both the EFS and the OS.

In addition, we examined the clinical outcomes of patients based on p-p38 expression by the Kaplan-Meier analysis. The 6-year EFS was 83 % for p-p38^−^ tumor tissues and 33 % for p-p38^+^ tumor tissues (*p* = 0.001) (Fig. 3c). The 5-year OS was 74 % for the p-p38^−^ tumor tissues vs 58 % for the p-p38^+^ tumor tissues (*p* < 0.001) (Fig. 3d).

Overall, the above findings demonstrated the impact of not only for p38 but also for p-p38 expression in tumor tissues on both the EFS and the OS.

### Relationship between the expression of p38 or p-p38 and the expression of Bcl-2 on clinical outcomes

Bcl-2 expression was also determined in this study. Consistent with the literature, 37 % of the 80 cases that were analyzed for Bcl-2 were scored as positive [26] (Table 2). There were no significant differences in the percentages of patients with elevated LDH (45 % vs 52 %), High IPI (56 % vs 68 %), median age (*p* = 0.6), stage III-IV (*p* = 0.5), when compared according to Bcl-2 expression (data no shown). Interestingly, the performance status >1 showed significant differences between Bcl-2 positive and negative subgroups (*p* = 0.05), similar to the p38 and p-p38 expressions (data no sown).

Several studies have reported the prognostic significance of Bcl-2 in DLBCL [25]. Accordingly, we have determined the correlation between p-p38 and Blc-2 expressions, based on our previous in vitro results, whereby the p38 MAPK expression correlated with Bcl-2 expression and the inhibition of either p38 or Bcl-2-induced chemosensitization to drugs [15].

We analyzed the relationship between the co-expression of the p38 or p-p38 and Bcl-2 expression in tumor tissues. All of the tissues that were p-p38^+^ were also Bcl-2^+^. Interestingly, 90 % of p-p38^−^ tissues were also Bcl-2^−^. With respect to p38 expression, 44 % of the p38^+^ tumor tissues were Bcl-2^+^ (*p* < 0.005). Noteworthy, tumor tissues that were p38^−^ were also Bcl-2^−^ (*p* = 0.01) (Table 2).

**Fig. 4:**
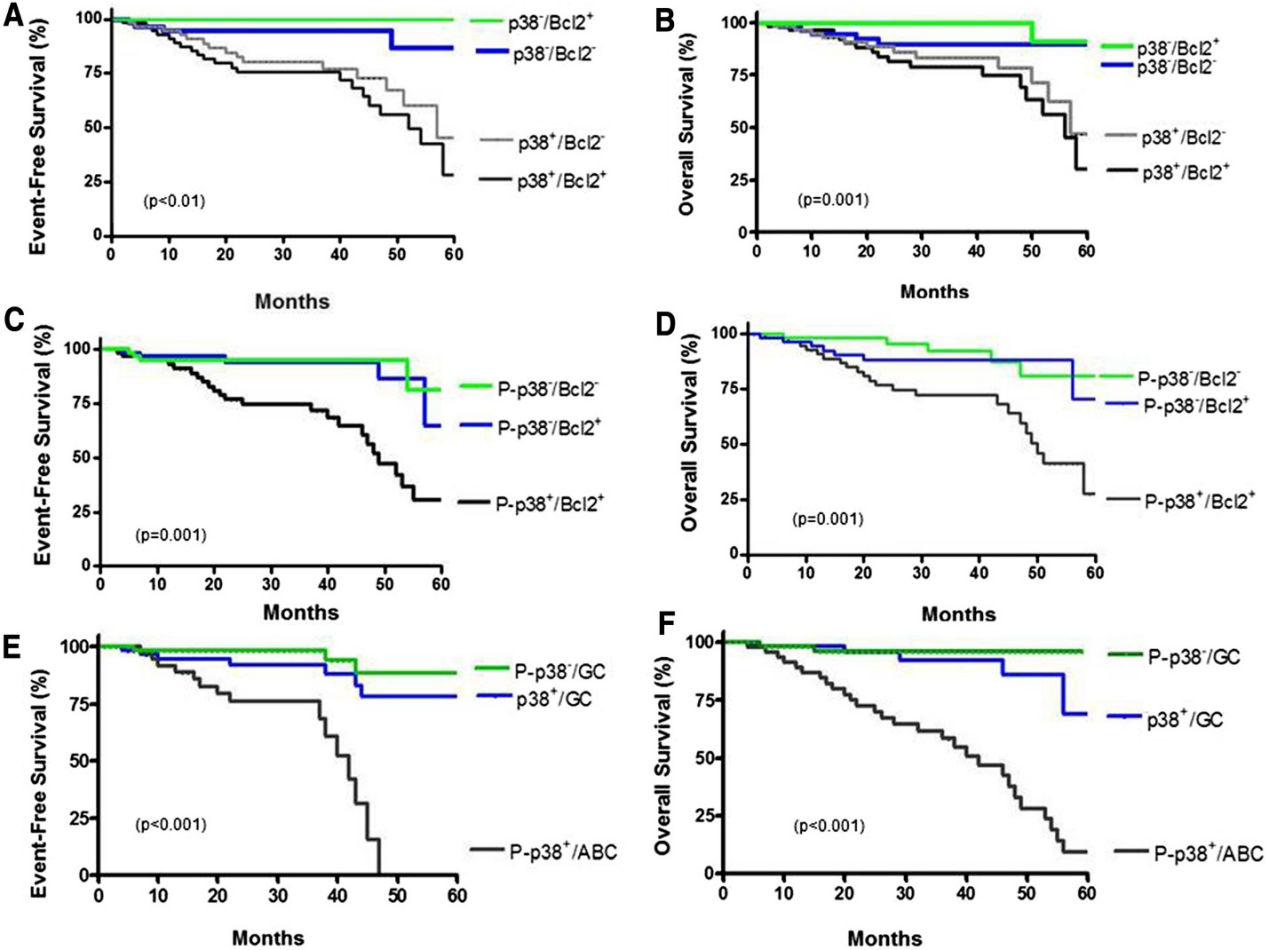
Clinical outcomes according to p-p38 MAPK, Bcl-2 expression and the DLBCL phenotype. **a** Event-free survival (EFS) curves for DLBCL patients treated with CHOP according to p38 MAPK and Bcl-2 expressions. **b**. Overall survival (OS) curves for DLBCL patients treated with CHOP according to p38 MAPK and Bcl-2 expressions. The p38 MAPK negative and Bcl-2 negative subgroups had more favorable PFS and OS than did the other two groups. **c** EFS curves according to the p-p38 MAPK and Bcl-2 expressions. **d** OS curves for DLBCL patients treated with CHOP according to p-p38 MAPK and Bcl-2 expressions. The p-p38 MAPK negative and Bcl-2 negative or positive subgroups had more favorable PFS and OS than did the other group. **e** EFS curves according to the p38 MAPK expression, p-p38 MAPK expression and the GC or ABC subtype. **f** Overall survival (OS) curve for DLBCL patients treated with CHOP according to p38 MAPK expression, p-p38 MAPK expression and the GC or ABC subtype. The p38 MAPK or p-p38 MAPK negative and GC subgroups had more favorable PFS and OS than did the other two groups

The clinical outcome of tumor tissues co-expressing p38/p-p38 and Bcl-2 were analyzed by Kaplan Meier (Fig. 4). The five-year EFS scores were 28 %, 45 %, 100 %, and 86 % for the p38^+^/Bcl-2^+^, p38^+^/Bcl2^−^, p38^−^/Bcl-2^+^, p38^−^/Bcl-2^−^ subgroups, respectively (Fig. 4a). In comparison with the other subgroups, there was a lower risk of EFS for the p38^−^/Bcl-2^+^ and p38^−^/Bcl-2^−^ tissues (*p* = 0.004). When the p-p38 expression positivity was considered, the five-year EFS scores were 30.6 %, 64.5 %, and 81 % for the p-p38^+^/Bcl-2^+^, p-p38^−^/Bcl-2^+^, p-p38^−^/Bcl-2^−^, respectively, and the p-p38^+^/Bcl-2^−^ subgroup was not detected (Fig. 4c). By comparison, we observed a lower risk of EFS for the p-p38^−^/Bcl-2^−^ and p-p38^−^/Bcl-2^+^ subgroups (*p* = 0.001). The five-year OS rates for p38 expression were 30 %, 46.7 %, 90.9 %, and 89.9 % for the p38^+^/Bcl-2^+^, p38^+^/Bcl-2^−^, p38^−^/Bcl-2^+^, p38^−^/Bcl-2^−^ subgroups, respectively (Fig. 4b). Patients’ tumors that were p38^−^/Bcl-2^−^ and p38^−^/Bcl-2^+^ had higher OS than those in the other two subgroups (*p* = 0.001). The five-year OS rates were 30.6 %, 64.5 %, and 81 % for the p-p38^+^/Bcl-2^+^, p-p38^−^/Bcl-2^+^, p-p38^−^/Bcl-2^−^ subgroups, respectively, and p-p38^+^/Bcl-2^−^ was not detected (Fig. 4d). Tumor tissues from patients showing p-p38^−^/Bcl-2^−^ and p-p38^−^/Bcl-2^+^ had higher OS than the other two subgroups (*p* = 0.001). According to observations reported in other studies [23], the differences in response rates were noted according to Bcl-2 expression (*p* = 0.005), EFS (*p* = 0.05) or OS (*p* = 0.005), similar to those observed with p-p38 expression.

In addition, we compared the survival curves for subgroups based on their co-expression of p38, p-p38 and the GC/ABC subtypes available in the cohort of this study. The 5-year EFS scores for the p38^+^/GC, p-p38^+^/ABC, and p-p38^−^/GC subgroups, were 78.3 %, 88.6 %, and 0 %, respectively (Fig. 4e). Patients in the subgroups p38^+^/GC and p-p38^−^/ GC had a lower risk of recurrence than the other two subgroups (*p* < 0.001). For the OS for 5 years, the scores were 69 %, 95.9 %, and 9.2 %, respectively (Fig. 4f). The patients in the groups p38^+^/GC and p-p38^−^/GC showed a higher OS than the other subgroups (*p* < 0.001). Similar to the expression of Bcl-2, the ABC subtype correlated with p-p38 expression and had poor EFS and OS. Comparing GC and ABC subgroups, there were marginal differences in the percentages of patients with elevated LDH (55 % vs 62 %), High IPI (64 % vs 70 %), stage III-IV (44 % vs 53 %), with the exception of a marginal higher percentage of performance status >1 (43 % vs 74 %). Similar to a previously reported study [27] the differences in response rates were observed (*p* = 0.005), EFS (*p* = 0.001) or OS (*p* = 0.005) and were noted according to the GC vs ABC phenotypes (data not shown).

The above findings demonstrated that the co-expressions of Bcl-2 and p-p38 are an indicator of poor EFS and OS. Further, the co-expression of Bcl-2 with p-p38 correlated with low EFS scores and poor OS for the patient’s tumor with the ABC subtype.

### Prognostic impact of p-p38 expression

We examined the prognostic impact of tumor tissues expressing p-p38 as a biomarker with respect to CHOP therapy. Cox multivariate analyses were performed for the p-p38 positivity in the R and NR subgroups and adjusted for the Bcl-2 and the immunophenotype categorization. As shown in Table 3, the expression of p-p38 had a significant prognostic value for both EFS and OS with an association to the ABC subtype and Bcl-2 expression. Multivariate analyses showed p-p38 expression (*p* < 0.05, HR = 2.54, 95 % CI 1.01–6.4), Bcl-2 expression (*p* < 0.05, HR = 2.26, 95 % CI 1.04–7.21) and the ABC subtype (*p* < 0.05, HR = 1.69, 95 % CI 1.07–3.63) to be independent factors associated with EFS (Table 3). Multivarite Cox analyses also indicated that p-p38 expression (*p* < 0.05, HR = 2.93, 95 % CI 1.13–7.5) and Bcl-2 expression (*p* < 0.05, HR = 3.33, 95 % confidence interval 1.28–6.63) and the ABC subtype (*p* < 0.05, HR = 2.45, 95 % CI 1.68–4.56) to be independent factors associated with OS (Table 3). When the relative risk was estimated with respect to the p-p38 expression after adjusting for IPI and Bcl-2 expressions, the p-p38 status in combination with high IPI and Bcl-2 expression were determinants of EFS (*p* < 0.05) and death (*p* < 0.05), (Additional file 3: Table S2). These data identified Bcl-2 expression, the ABC subtype and p-p38 expression as significant risk factors for inferior EFS and OS in DLBCL lymphoma. Noteworthy, the expressions of both p38 p-p38 remained as prognostic factors even upon adjustments for both the Bcl-2 expression and the ABC categorization.

**Table 3:** Multivariate analysis of risk factors of EFS and OS of DLBCL patients treated with CHOP

	Relative risk	95 % Cl	*P*
EFS			
P-p38^+^	2.54	1.01–6.4	<0.05
Bcl-2^+^	2.26	1.04–7.21	<0.05
ABC	1.69	1.07–3.63	<0.05
OS			
P-p38^+^	2.93	1.13–7.5	<0.05
Bcl-2^+^	3.33	1.28–6.63	<0.05
ABC	2.45	1.68–4.56	<0.05

*Cl* Confidence intervals, *EFS* event-free survival, *OS* overall survival

## Discussion

The present study demonstrates a significant correlation between the expression of p38 and p-p38 MAPK and the development of resistance to CHOP treatment in patients with DLBCL. The patient population studied in this report was primarily treated with CHOP only and rituximab was not an option for these patients. The p-p38 MAPK expression is increased in clinical tissue samples from untreated patients who were found subsequently to be resistant to CHOP chemotherapy. Kaplan Meier showed that patients’ tumor tissues expressing p38 or p-p38 experienced reduced EFS and shorter OS compared to patients’ tumor tissues with no expression of p38 or p-p38 and experienced longer EFS and OS. In addition, there was a significant correlation between the expression of Bcl-2 and p-p38, however, the EFS and OS correlated primarily with only the expression of p38 or p-p38 irrespective of Bcl-2. In addition, we show here that the expression of either p38 or p-p38 MAPK is an independent prognostic biomarker from the expression of Bcl-2.

The role of p38 MAPK in cancer depends on the cell type and cancer stage. Some studies have reported that it plays a role as an oncogene whereas in other studies the expression of p38 MAPK is described as a tumor suppressor. For example, p38α expression is down regulated in lung tumors, suggesting that p38 loss could be related to tumor formation [12]. Consistent with these findings, the expression of MKK4, a p38 activator, is reduced in advanced tumors [28]. Conversely, increased levels of p38α have been correlated with malignancy in follicular lymphoma, lung, thyroid, breast carcinoma, glioma, and head and neck squamous cell carcinoma [14]. In colorectal cancer, p38α is required for cell proliferation and survival and its inhibition leads to cell-cycle arrest and autophagic cell death [16]. In addition, higher level of p-p38 in colon tumor samples from patients correlated with refractoriness to irinotecan-based therapy [29]. p38 activation contributes to tamoxifen resistance in ER-positive breast tumors [30]. More recently, it has been shown that p38 inhibition enhances the sensitivity to arsenic trioxide and bortezomib in multiple myeloma [31]. Malignant B cells from SLL/CLL patients had higher basal levels of p-p38 compared to healthy donor B cells [32].

Here we show an impact of p38 and p-p38 expressions on the prognosis of DLBCL that has not been previously reported. The findings showed a worse EFS and OS in patients tumors with p38^+^ and p-p38^+^ compared with patients tumors with p38^−^ and p-p38^−^. In our cohort, the expression of single p38^+^ or p-p 38^+^ had an impact on survival, and its association with poor PFS. These findings suggested that p38 and p-p38 expressions had an unfavorable prognostic effect in patients with DLBCL.

Previous related studies showed increased expression of the p38 MAPK transcript and protein in a subset of DLBCL arising from follicular lymphomas [33]. Similar observations were obtained by Ogasawara et al. [34] who found that freshly prepared cells from B cell lymphomas showed constitutive in vitro activity of p38 MAPK and ERK but not *Ras*, Akt kinase, and JNK kinase. These observations suggest that the constitutive activation of the p38 MAPK pathway may be implicated in lymphomagenesis.

The high p-p38 MAPK expression in reported studies and in the present study may be reflective of the presence of cytokines or growth factors that activate the p38 MAPK pathway in these tumors. Reported studies have indicated that the constitutive activation of the p38 MAPK pathway may be regulated by IL-4 with stimulatory effects on gene expression, protein levels, and in vitro kinase activity. These data suggested a possible role for IL-4-mediated activation of the p38 MAPK signaling pathway in the pathogenesis of B cell lymphomas [35].

We have found that 100 % of p-p38 MAPK positive tumors were also positive for Bcl-2 expression (Table 2). Bcl-2 is an anti-apoptotic protein involved in the regulation of programmed cell death [36]. Bcl-2 overexpression was associated with poorer survival in the pre-rituximab era [37]. In contrast, Bcl-2 expression was not identified as a prognostic factor by Hans et al. [22]. However, several other studies have reported the prognostic value of Bcl-2 expression in patients with DLBCL [37, 38]. This association of Bcl-2 expression with survival in DLBCL patients treated with CHOP or CHOP-like regimens had conflicting results [39]. Conflicts about the prognostic significance of Bcl-2 expression have been attributed to the heterogeneity of DLBCL. Recent studies reported that the controversial results can be explained in the context of the cell origin of DLBCL. Bcl-2 overexpression in ABC-DLBCL patients treated with CHOP regimens have poor prognosis [39], probably due to the tumor cells’ ability to resist apoptosis induced by chemotherapy. The addition of rituximab to CHOP regimens for DLBCL has led to a significant increase in patient’s survival and is now considered a standard regimen for DLBCL. Controversial results have also been reported for the association of Bcl-2 expression in R-CHOP-treated cohorts; in general, Bcl-2 expression influences the relative risk in OS in Bcl-6^+^ DLBCL but not in the Bcl-6^−^ cases [37]. The inclusion of the expression and activation of p38 MAPK as diagnostic markers can limit the heterogeneity of this subgroup of patients. It has been suggested that the expression and activity of p38 MAPK may be considered as prognostic factors and can lead for the choice of appropriate treatment regimens [40]. According to our previous *in vitro* results [15] and clinically extended studies [41, 42], it was suggested that patient’s tumors with p38 MAPK activation and which are Bcl-2 positive reflected a chemoresistant phenotype. Such patients may respond to chemotherapy used in combination with rituximab since our *in vivo* and *in vitro* studies revealed that the rituximab treatment inhibits the p38 MAPK/Bcl-2 pathway [15] and sensitizes the resistant tumor cells to drug-induced apoptosis. However, patients were not responding to CHOP and don’t have the treatment option with rituximab, we suggest that inhibitors of the p38 MAPK may reverse resistance.

Due to our present findings that all tumors that are positive for p-p38 expression are also positive for Bcl-2, the relationship between p38 activation and Bcl-2 expression may be mediated by transcriptional and post transcriptional mechanisms, which may affect the anti-apoptotic Bcl-2 family proteins [43]. We and other have reported that p38 MAPK can mediate the transcriptional expression of IL-10 via the Sp1 transcription factor and upon IL-10 interaction with its cognate receptors it induces the activation of Stat-3 and Bcl-2 expression [15, 44]. It is known that the interaction between Bcl-2 and p38 MAPK can directly regulate either its activity or indirectly via the transcriptional regulation through NF-κB activation [45].

NF-κB is centrally involved in lymphomagenesis and tumor progression in various types of lymphoma. Over-expression of NF-κB in tumor tissues has previously been observed in NHL [46]. Recent studies have confirmed that NF-κB expression was predominantly seen in ABC-DLBCL [47]. Our previous findings have implicated the NF-κB pathway in the p38 MAPK/Bcl-2 circuit of chemoresistance [15]. However, in this study we did not find a correlation between the clinical features for p38 MAPK expression and NF-κB expression. We have further shown that 54 % of p-p38 MAPK^+^ are positive for NF-κB, while the remaining 46 % of those patients were negative for NF-κB (Table 2). Previous studies have shown that ABC-DLBCL tumors exhibit higher expression levels of NF-κB target genes than those found in the GBC subtype [47]. The constitutive NF-κB signaling pathway confers a chemoresistant phenotype in NHL cell lines [46]. Treatment with either specific chemical inhibitors or with rituximab reverses chemoresistance or results in the selective cytotoxicity in NHL or ABC-DLBCL cell lines, respectively [48]. It has been shown that the p38 MAPK pathway is involved in the activation of NF-κB through the phosphorylation of MSK 1/2 directly or mediated through H3 phosphorylation [49]. However, the present findings did not establish a roll of NFκB in the response of CHOP. We suggest that the activation of p38 MAPK may be the result of other regulatory mechanisms.

When we analyzed several clinical parameters for the p38 and p-p38 MAPK expressions, we have found no significant differences among the four subgroups with regards to gender, age disease stage, LDH levels, and the IPI score. In contrast, poor performance status was the only dependent risk factor for p-p38 MAPK expression in our DLBCL cohort. Several clinical parameters such as elevated serum, LDH levels and extranodal or bone marrow involvement have been established as risk factors for relapse in DLBCL [50]. The performance status is measured by a performance score that takes into account several factors. We cannot establish if poor PS and p-p38 MAPK expression have a correlation with OS in our cohort. However, we have analyzed the correlation between the expressions of p38, p-p38 MAPK and the DLBCL subtype. Interesting, our results showed that the ABC subtype correlates with p-p38 expression. Multivariate analysis confirmed that the ABC subtype with p-p38 expression is associated with unfavorable EFS and OS.

## Conclusions

Altogether, the present findings show that activated p-p38 was expressed in 30 % of DLBCL and such an expression was associated with the ABC phenotype and poor survival. In addition, p38 expression was associated with no response to the CHOP regimen. Considering the potential prognostic and therapeutic roles of p38 and p-p38 MAPK, we suggest their inclusion as additional biomarkers for diagnosis in untreated tumor specimens. The positive expression predicts failure to CHOP treatment alone in a subset of patients and it is recommended that a combination therapy with R-CHOP be considered for this subset of patients or the use of inhibitors of p38 MAPK. In addition, from the potential prognostic utility of p38 MAPK expression, the findings reported herein may help future investigations to determine the clinical usefulness of specific chemical inhibitors of the p38 MAPK pathway in the treatment of DLBCL in combination with conventional CHOP regimens.

## Additional files

Additional file 1: Table S1. Source of antibodies and dilutions used (JPEG 47 kb)
Additional file 2: Figure S1. Expression of p65 and p50 (NF-κB) in patients with DLBCL. Tissue microarray-based immunohistochemical analyses of p50 and p65 in representative tumor biopsies from DLBCL patients. DLBCL array cores showing over-expression of p50 (A) and high expression of p65 (B). 40X objective on an Olympus BX 51 microscope (Olympus America, Center Valley, PA, USA), (Left) and 100X aperture view of the same tissue (Right). Of the 80 DLBCL biopsies analyzed, 16 % were positive for p50 and 32 % were positive for p65 (C). (JPEG 75 kb)
Additional file 3: Table S2. Multivariate analysis of relative risk estimates for p-p38^+^ vs p-p38^−^ expression in DLBCL patients treated with CHOP adjusting for IPI and Bcl-2 expression. (JPEG 55 kb)

## Abbreviations

ABC: Activated B-Cell subtype
B-NHL: B cell No-Hodgkin Lymphoma
CHOP-R: CHOP-Rituximab
DLBCL: Diffussed Large B-Cell Lymphoma
EFS: Event-free survival
GCB: Germinal Center B-Cell subype
IHC: Immunohistochemistry
IPI: The International Prognostic Index
LDH: Lactate Dehydrogenase
MAPK: Mitogen-Activated Protein Kinase
MUM-1: Multiple Myeloma Oncogene 1
NF-κB: Nuclear Factor Kappa-Light-Chain-Enhancer of Activated B cells
OS: Overall Survival
P53: Tumor Protein 53
REAL: Revised European American Lymphoma Classification
TMA: Tissue Microarray

## References

[1] Staudt LM (2010). Aggressive lymphoma. N. Engl J Med.

[2] Siegel R, Ward E, Brawley O, Jemal A (2011). Cancer statistics, 2011: the impact of eliminating socioeconomic and racial disparities on premature cancer deaths. CA Cancer J Clin.

[3] Flowers CR, Shenoy PJ, Borate U, Bumpers K, Douglas-Holland T, King N (2013). Examining Racial Differences in Diffuse Large B-Cell Lymphoma Presentation and Survival. Leuk Lymphoma.

[4] Perry AM, Cardesa-Salzmann TM, Meyer PN, Colomo L, Smith LM, Fu K (2012). A new biologic prognostic model based on immunohistochemistry predicts survival in patients with diffuse large B-cell lymphoma. Blood..

[5] Sehn LH, Berry B, Chhanabhai M, Fitzgerald C, Gill K, Hoskins P (2007). The revised International Prognostic Index (R-IPI) is a better predictor of outcome than the standard IPI for patients with diffuse large B-cell lymphoma treated with R-CHOP. Blood.

[6] Choi WW, Weisenburger DD, Greiner TC, Piris MA, Banham AH, Delabie J (2009). A new immunostain algorithm classifies diffuse large B-cell lymphoma into molecular subtypes with high accuracy. Clin Cancer Res..

[7] Nyman H, Jantunen E, Juvonen E, Elonen E, Böhm J, Kosma VM (2008). Impact of germinal center and non-germinal center phenotypes on overall and failure-free survival after high-dose chemotherapy and auto-SCT in primary diffuse large B-cell lymphoma. Bone Marrow Transplant..

[8] Nyman H, Adde M, Karjalainen-Lindsberg ML, Taskinen M, Berglund M, Amini RM (2007). Prognostic impact of immunohistochemically defined germinal center phenotype in diffuse large B-cell lymphoma patients treated with immunochemotherapy. Blood.

[9] Menon MP, Pittaluga S, Jaffe ES (2012). The histological and biological spectrum of diffuse large B-cell lymphoma in the World Health Organization classification. Cancer J.

[10] Cuadrado A, Nebreda AR (2010). Mechanisms and functions of p38 MAPK signalling. Biochem J..

[11] Cuenda A, Rousseau S (2007). p38 MAP-kinases pathway regulation, function and role in human diseases. Biochim Biophys Acta.

[12] Ventura JJ, Tenbaum S, Perdiguero E, Huth M, Guerra C, Barbacid M (2007). p38alpha MAP kinase is essential in lung stem and progenitor cell proliferation and differentiation. Nat Genet.

[13] Yoshizuka N, Chen RM, Xu Z, Liao R, Hong L, Hu WY (2012). A novel function of p38-regulated/activated kinase in endothelial cell migration and tumor angiogenesis. Mol Cell Biol.

[14] Wagner EF, Nebreda AR (2009). Signal integration by JNK and p38 MAPK pathways in cancer development. Nat Rev Cancer.

[15] Vega MI, Huerta-Yepaz S, Garban H, Jazirehi A, Emmanouilides C, Bonavida B (2004). Rituximab inhibits p38 MAPK activity in 2 F7 B NHL and decreases IL-10 transcription: pivotal role of p38 MAPK in drug resistance. Oncogene.

[16] Chiacchiera F, Simone C (2008). Signal-dependent regulation of gene expression as a target for cancer treatment: inhibiting p38alpha in colorectal tumors. Cancer Lett.

[17] Olson JM, Hallahan AR (2004). p38 MAP kinase: a convergence point in cancer therapy. Trends Mol Med.

[18] Pfreundschuh M, Schubert J, Ziepert M, Schmits R, Mohren M, Lengfelder E (2008). Six versus eight cycles of bi-weekly CHOP-14 with or without rituximab in elderly patients with aggressive CD20+ B-cell lymphomas: a randomised controlled trial (RICOVER-60). Lancet Oncol.

[19] Carbone PP, Kaplan HS, Musshoff K, Smithers DW, Tubiana M (1971). Report of the Committee on Hodgkin’s Disease Staging Classification. Cancer Res.

[20] Oken MM, Creech RH, Tormey DC, Horton J, Davis TE, McFadden ET, Carbone PP (1982). Toxicity and response criteria of the Eastern Cooperative Oncology Group. Am J Clin Oncol..

[21] Zinzani PL, Broccoli A, Stefoni V, Musuraca G, Abruzzese E, De RA (2010). Immunophenotype and intermediate-high international prognostic index score are prognostic factors for therapy in diffuse large B-cell lymphoma patients. Cancer.

[22] Hans CP, Weisenburger DD, Greiner TC, Gascoyne RD, Delabie J, Ott G (2004). Confirmation of the molecular classification of diffuse large B-cell lymphoma by immunohistochemistry using a tissue microarray. Blood.

[23] Iqbal J, Neppalli VT, Wright G, Dave BJ, Horsman DE, Rosenwald A (2006). BCL2 expression is a prognostic marker for the activated B-cell-like type of diffuse large B-cell lymphoma. J Clin Oncol.

[24] Bavi P, Uddin S, Bu R, Ahmed M, Abubaker J, Balde V (2011). The biological and clinical impact of inhibition of NF-kappaB-initiated apoptosis in diffuse large B cell lymphoma (DLBCL). J Pathol.

[25] Kaplan EL, Meier P (1958). Nonparametric estimation from incomplete observations. J Am Statist Assoc..

[26] Mounier N, Briere J, Gisselbrecht C, Emile JF, Lederlin P, Sebban C (2003). Rituximab plus CHOP (R-CHOP) overcomes bcl-2--associated resistance to chemotherapy in elderly patients with diffuse large B-cell lymphoma (DLBCL). Blood..

[27] Kubuschok B, Held G, Pfreundschuh M (2015). Management of Diffuse Large B-Cell Lymphoma (DLBCL). Cancer Treat Res..

[28] Robinson VL, Hickson JA, Vander Griend DJ, Dubauskas Z, Rinker-Schaeffer CW (2003). MKK4 and metastasis suppression: a marriage of signal transduction and metastasis research. Clin Exp Metastasis.

[29] Paillas S, Boissiere F, Bibeau F, Denouel A, Mollevi C, Causse A (2011). Targeting the p38 MAPK pathway inhibits irinotecan resistance in colon adenocarcinoma. Cancer Res.

[30] Gutierrez MC, Detre S, Johnston S, Mohsin SK, Shou J, Allred DC (2005). Molecular changes in tamoxifen-resistant breast cancer: relationship between estrogen receptor, HER-2, and p38 mitogen-activated protein kinase. J Clin Oncol.

[31] Wen J, Feng Y, Huang W, Chen H, Liao B, Rice L (2010). Enhanced antimyeloma cytotoxicity by the combination of arsenic trioxide and bortezomib is further potentiated by p38 MAPK inhibition. Leuk Res.

[32] Blix ES, Irish JM, Husebekk A, Delabie J, Forfang L, Tierens AM (2012). Phospho-specific flow cytometry identifies aberrant signaling in indolent B-cell lymphoma. BMC Cancer.

[33] Elenitoba-Johnson KS, Jenson SD, Abbott RT, Palais RA, Bohling SD, Lin Z (2003). Involvement of multiple signaling pathways in follicular lymphoma transformation: p38-mitogen-activated protein kinase as a target for therapy. Proc Natl Acad Sci U S A..

[34] Ogasawara T, Yasuyama M, Kawauchi K (2003). Constitutive activation of extracellular signal-regulated kinase and p38 mitogen-activated protein kinase in B-cell lymphoproliferative disorders. Int J Hematol..

[35] Ding H, Gabali AM, Jenson SD, Lim MS, Elenitoba-Johnson KS (2009). P38 mitogen activated protein kinase expression and regulation by interleukin-4 in human B cell non-Hodgkin lymphomas. J Hematop..

[36] Merino R, Ding L, Veis DJ, Korsmeyer SJ, Nunez G (1994). Developmental regulation of the Bcl-2 protein and susceptibility to cell death in B lymphocytes. EMBO J.

[37] Iqbal J, Meyer PN, Smith LM, Johnson NA, Vose JM, Greiner TC (2011). BCL2 predicts survival in germinal center B-cell-like diffuse large B-cell lymphoma treated with CHOP-like therapy and rituximab. Clin Cancer Res.

[38] Martin-Arruti M, Vaquero M, Díaz de Otazu R, Zabalza I, Ballesteros J, Roncador G (2012). Bcl-2 and BLIMP-1 expression predict worse prognosis in gastric diffuse large B cell lymphoma (DLCBL) while other markers for nodal DLBCL are not useful. Histopathology.

[39] Dunleavy K, Wilson WH (2011). Differential role of BCL2 in molecular subtypes of diffuse large B-cell lymphoma. Clin Cancer Res.

[40] Handra-Luca A, Lesty C, Hammel P, Sauvanet A, Rebours V, Martin A (2012). Biological and prognostic relevance of mitogen-activated protein kinases in pancreatic adenocarcinoma. Pancreas.

[41] Vega M, Martinez-Paniagua M, Sanchez-Arellano B, Martinez-Miguel B, vilez-Salas A, Hernandez-Pando R, et al. Activation of the p38MAPK signaling pathway and over-expression of Bcl-2/Bcl-XL in patients with DLBCL: potential biomarkers and targets for therapeutics. A Mexican Lymphoma Group Study. Cancer Res. 2007;67:723.

[42] Vega MI, Huerta-Yepez S, Martinez-Paniagua MA, Bonilla CG, Bonavida B (2006). Rituximab-Mediated Inhibition of the p38 MAPK and NF-{kappa}B Survival Pathways In Vitro and Validation in Nude Mice Bearing the Raji B-NHL Tumor. Blood.

[43] Cargnello M, Roux PP (2011). Activation and function of the MAPKs and their substrates, the MAPK-activated protein kinases. Microbiol Mol Biol Rev.

[44] Ma W, Lim W, Gee K, Aucoin S, Nandan D, Kozlowski M (2001). The p38 mitogen-activated kinase pathway regulates the human interleukin-10 promoter via the activation of Sp1 transcription factor in lipopolysaccharide-stimulated human macrophages. J Biol Chem.

[45] Arthur JS (2008). MSK activation and physiological roles. Front Biosci.

[46] Baldwin AS (2001). Control of oncogenesis and cancer therapy resistance by the transcription factor NF-kappaB. J Clin Invest.

[47] Davis RE, Brown KD, Siebenlist U, Staudt LM (2001). Constitutive nuclear factor kappaB activity is required for survival of activated B cell-like diffuse large B cell lymphoma cells. J Exp Med.

[48] Vega MI, Martinez-Paniagua M, Jazirehi AR, Huerta-Yepez S, Umezawa K, Martinez-Maza O (2008). The NF-kappaB inhibitors (bortezomib and DHMEQ) sensitise rituximab-resistant AIDS-B-non-Hodgkin lymphoma to apoptosis by various chemotherapeutic drugs. Leuk Lymphoma.

[49] Saccani S, Pantano S, Natoli G (2002). p38-Dependent marking of inflammatory genes for increased NF-kappa B recruitment. Nat Immunol.

[50] Villa D, Connors JM, Shenkier TN, Gascoyne RD, Sehn LH, Savage KJ (2010). Incidence and risk factors for central nervous system relapse in patients with diffuse large B-cell lymphoma: the impact of the addition of rituximab to CHOP chemotherapy. Ann Oncol.

