# Supplementary figures and images for: P38 MAPK expression and activation predicts failure of response to CHOP in patients with Diffuse Large B-Cell Lymphoma
Source: BMC Cancer. 2015 Oct 16;15:722. doi: 10.1186/s12885-015-1778-8 (PMC4609122; doi:10.1186/s12885-015-1778-8)

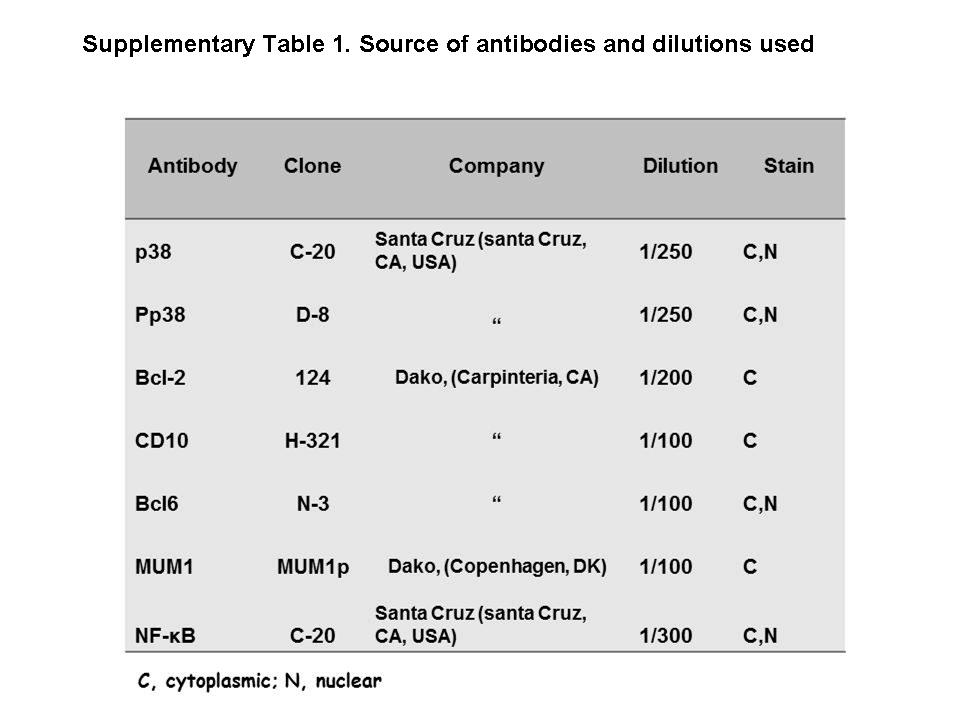

Supplement: Additional file 1: Table S1. — Source of antibodies and dilutions used (JPEG 47 kb) [file 12885_2015_1778_MOESM1_ESM.jpeg]

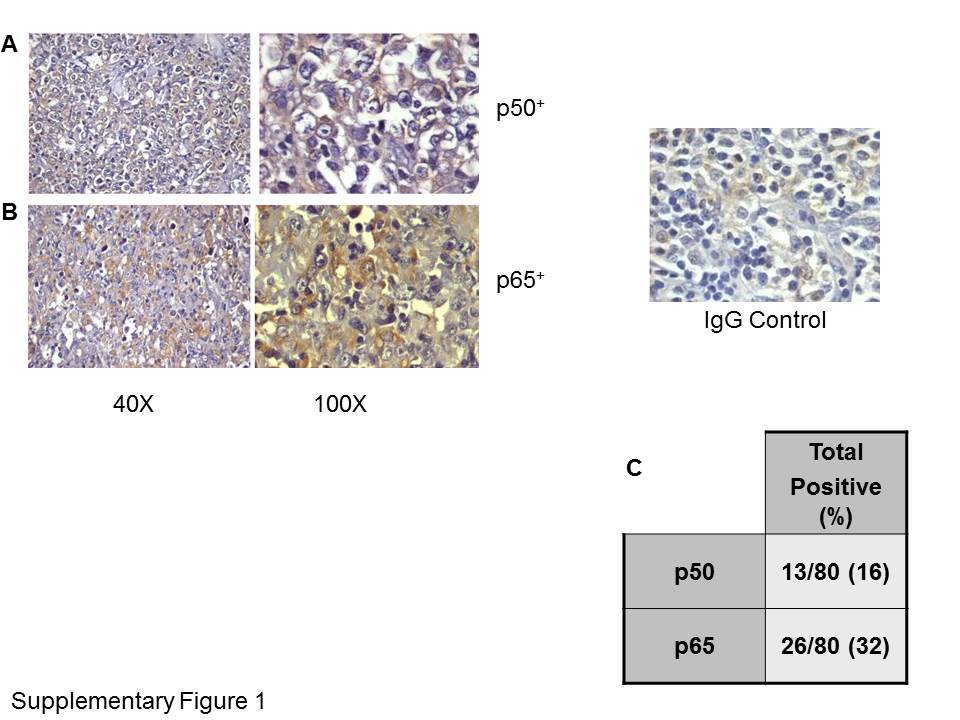

Supplement: Additional file 2: Figure S1. — Expression of p65 and p50 (NF-κB) in patients with DLBCL. Tissue microarray-based immunohistochemical analyses of p50 and p65 in representative tumor biopsies from DLBCL patients. DLBCL array cores showing over-expression of p50 (A) and high expression of p65 (B). 40X objective on an Olympus BX 51 microscope (Olympus America, Center Valley, PA, USA), (Left) and 100X aperture view of the same tissue (Right). Of the 80 DLBCL biopsies analyzed, 16 % were positive for p50 and 32 % were positive for p65 (C). (JPEG 75 kb) [file 12885_2015_1778_MOESM2_ESM.jpeg]

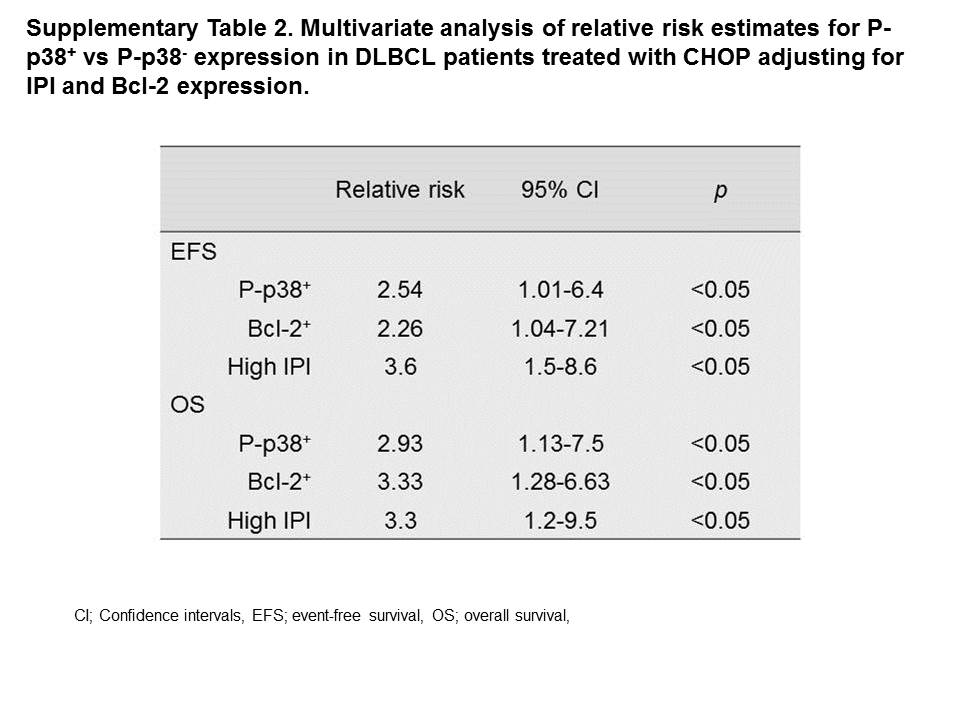

Supplement: Additional file 3: Table S2. — Multivariate analysis of relative risk estimates for p-p38+ vs p-p38− expression in DLBCL patients treated with CHOP adjusting for IPI and Bcl-2 expression. (JPEG 55 kb) [file 12885_2015_1778_MOESM3_ESM.jpeg]
